# Crystallization of high aspect ratio HKUST-1 thin films in nanoconfined channels for selective small molecule uptake†

**DOI:** 10.1039/c9na00254e

**Published:** 2019-06-12

**Authors:** Stephanie Guthrie, Luke Huelsenbeck, Armita Salahi, Walter Varhue, Natalie Smith, Xiaohan Yu, Lucy U. Yoon, Joshua J. Choi, Nathan Swami, Gaurav Giri

**Affiliations:** Department of Chemical Engineering, University of Virginia Charlottesville Virginia 22904 USA gg3qd@virginia.edu; Department of Electrical and Computer Engineering, University of Virginia Charlottesville Virginia 22904 USA; Department of Chemical Engineering, Tsinghua University Beijing 100084 China

## Abstract

We present the ability to create unique morphologies of a prototypical metal organic framework (MOF), HKUST-1, by carrying out its crystallization within a set of nano-confined fluidic channels. These channels are fabricated on cyclic olefin copolymer by the high-fidelity hot embossing imprinting method. The picoliter volume synthesis in the nanochannels is hypothesized to bias the balance between nucleation and growth rates to obtain high aspect ratio large-crystalline domains of HKUST-1, which are grown in defined morphologies due to the patterned nanochannels. Confined crystal growth is achieved in nanofluidic channels as shallow as 50 nm. HKUST-1 crystalline domains with aspect ratios greater than 2500, and lengths up to 144 μm are obtained using the nanochannels, exceeding values obtained using chemical modulation and other confinement methods. HKUST-1 crystals are characterized using optical microscopy and scanning electron microscopy with energy dispersive spectroscopy. Porosity of the MOF and selective molecular uptake is demonstrated through inclusion of anthracene and methylene blue within the HKUST-1 framework, and with exclusion of rhodamine B and riboflavin, characterized using a confocal fluorescence microscope. We attribute this selectivity to the analyte size and electrostatic characteristics. Nanoconfined crystallization of MOFs can thus yield control over crystalline morphology to create ideal MOF crystals for enabling selective molecular enrinchment and sensing.

## Abstract 2

High aspect ratio HKUST-1 MOF crystals are formed in a nanofluidic device, and utilized for selective molecular uptake.

## Graphical abstract

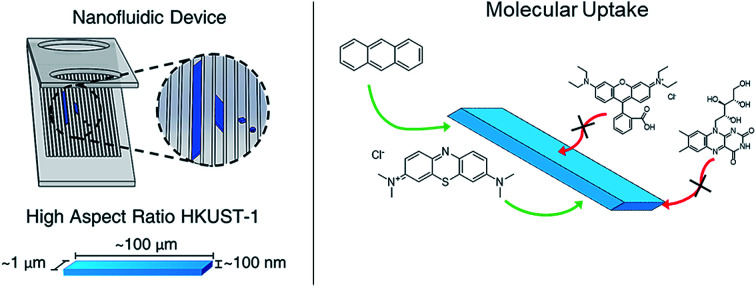

## Introduction

Metal–organic frameworks (MOFs) are composed of metal ions or oxo-metallic clusters coordinated with organic linkers. Highly porous, crystalline structures can be created with readily controlled pore geometry and chemistry by changing the metallic cluster or the organic linker.^1,2^ Due to their structural and chemical tunability, MOFs are useful for chemical storage, separations, electronic and photonic devices, catalysis, and many other applications.^3–7^

Several MOFs have been employed as molecular sieves to selectively enrich small molecules.^8–10^ Selective enrichment can be coupled with electrical or optical sensing techniques for detection of small molecules at low concentrations.^11,12^ The selectivity, enrichment and response time of MOF-based sensors are dependent on the analyte diffusion properties within the MOF. A variety of interactions can be utilized to influence adsorbate or analyte uptake and transport. For example, the adsorption capacity of the MOF MIL-101 for methyl orange, an anionic dye, was found to be enhanced by increasing the positive charge present on the MOF.^13^ MOF crystals with large aspect ratios and lowered defect concentrations have been shown to enhance charge and mass transport.^14,15^ Interaction between the guest molecule and the MOF can also be regulated by varying the MOF morphology. For example, a high aspect ratio MOF crystal can allow for selectivity due to predominating crystal facets that are accessible to the guest molecule (*e.g.* the {111} and {100} planes feature different electrostatic and chemical functionality that impacts analyte diffusion).^16^

Currently, MOF morphologies with large aspect ratios are not easily synthesized using bulk synthetic techniques. It is typical to control morphology through pre- or post-synthetic modifications (*e.g.* addition of surface directing agents).^17–19^ Several groups have utilized confined crystallization to control nucleation and growth of multiple material systems (including small organic molecules, perovskites and MOFs).^20–22^

The reduced volumes and small working dimensions in confined systems allow for precise regulation of heat and mass transport to modulate conditions during MOF formation.^20^ Recent approaches to confined crystallization in MOFs include interfacial synthesis and diffusion-mediated growth, providing control over MOF aspect ratio and creating oriented MOF thin films.^21–23^ Microstamping was used by Ameloot *et al.* to create nano-sized HKUST-1 single crystals in controlled patterns.^24^ Previous work has achieved MOF synthesis (ZIF-8, with aspect ratio 60) by growing nano-confined crystals inside anodized aluminum oxide or track etched polycarbonate membranes.^25^ Additionally, MOF nanosheets with aspect ratios of 1000 were formed using a biphasic synthesis method.^26^ A technique termed solution shearing can promote self-confined thin film growth in numerous organic molecules.^27–29^ Confinement based growth was also achieved by altering the wetting properties of a substrate to grow oriented, single crystals of an organic semiconductor, C8-BTBT.^30^ Perovskite single crystal domains were formed using ink transfer technology for geometric confinement during synthesis.^31^

Building from previous confinement techniques and applying nanofluidic crystallization technology to MOFs, the work presented here demonstrates the utility of nanochannel confinement-based crystallization to create high aspect ratio MOF crystals. In addition to improving MOF morphology control during synthesis, this study aims to lay the groundwork for nanofluidic small molecule enrichment using high aspect ratio MOFs.

We report a parallelized nanofluidic platform, fabricated *via* a hot-embossing nano imprint lithography method (HE NIL), for confined MOF crystal nucleation and growth. HE NIL can reliably replicate the nanoscale features of a master mold in an inexpensive wafer made of thermoplastic cyclic olefin copolymer (COC). We show that nano-confinement can aid in producing high aspect ratio, crystalline domains of a prototypical MOF, HKUST-1. Using an evaporation driven synthesis, HKUST-1 nucleation and growth occurs along the nanochannel length to yield high aspect ratio crystals. We observed crystal formation with aspect ratios up to 2500, exceeding that of any other previously reported MOFs to the best of the authors' knowledge.^32,33^ Finally, the capacity for selective molecular uptake and exclusion in high aspect ratio HKUST-1 crystals is demonstrated using anthracene, methylene blue, rhodamine B and riboflavin.

## Results and discussion

### Nanochannel fabrication and confined crystallization

**Fig. 1:**
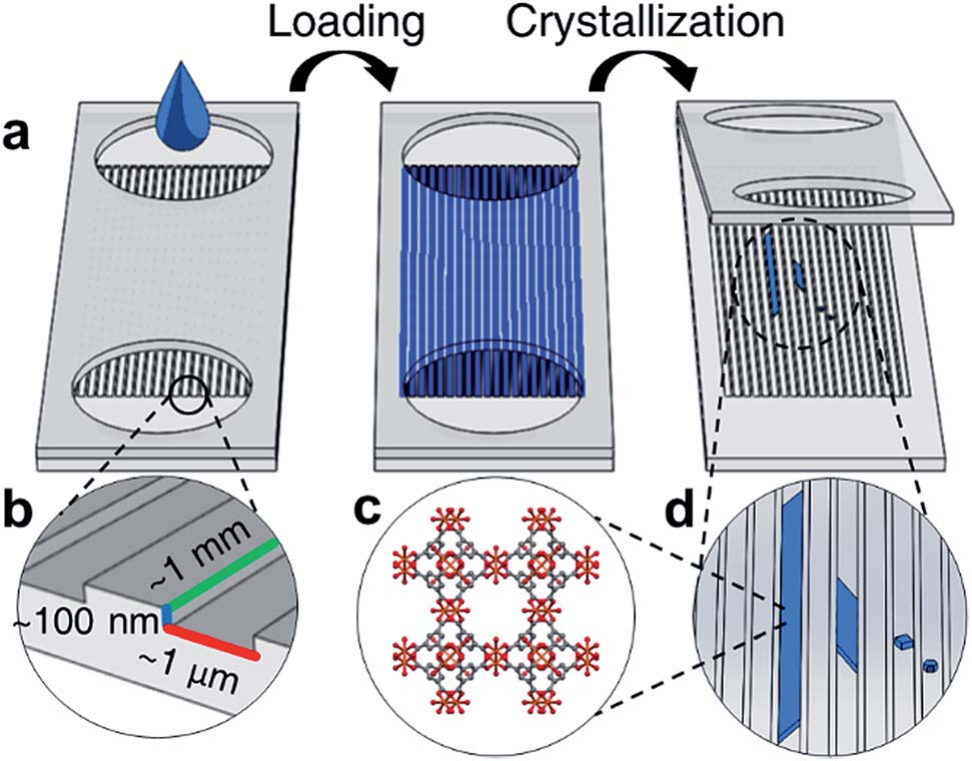
(a) Schematic of nanochannel loading through ports, followed by evaporation driven crystallization to obtain high aspect ratio MOF crystals (b) typical dimensions of fabricated nanochannels (c) molecular structure of HKUST-1 and (d) high aspect ratio HKUST-1 crystals grown in nanochannels.

Closed nanochannel arrays are created by the hot embossed bonding of two cyclic olefin copolymer (COC) substrates. The bottom substrate is patterned with nanochannel features that are transferred from a soft stamp embossing process (ESI Fig. 1†). The top COC plate contains microfluidic through-holes to serve as fluid inlet and outlets surrounding the enclosed channels (Fig. 1, ESI Fig. 2†).

Each nanochannel array contains a potential loading volume on the order of picoliters per nanochannel, and each array contains 50–100 channels. Channels with widths 0.5–5 μm, depths 60–350 nm, and lengths 2–5 mm were used in this study (ESI Tables 1 and 2†).

The nanochannels were loaded by placing a small volume of HKUST-1 precursor solution (5 μL) in the channel ports. Capillary action was observed to wick solution from the inlets, filling the void space in the empty channels. Using this technique most channels show fill efficiencies ranging from 40–70% (ESI Fig. 3†). Crystallization in the nanochannels is induced *via* solvent evaporation through a combination of vacuum application and heating.

As solvent evaporates from the channels, the solution front recedes at a rate of roughly 0.5 μm h^−1^, measured by observing the moving air-liquid interface from optical micrographs. The evaporation rate of solvent is slower than the diffusion rate of the precursors, therefore the spatial concentration of MOF precursor materials can be approximated to be uniform throughout the length of the channel.^34^ We hypothesize that during crystallization the concentration increase of the metal ion and the organic linker in the solution is uniform throughout the channel length, and heterogeneous HKUST-1 nucleation occurs throughout the length of the channel, rather than preferentially at the air-liquid interface. However, crystallization also occurs in clusters spanning several channels, suggesting that defects or localized surface properties of the nanochannels may promote nucleation.

Nanofluidic crystallization is able to reduce the occurrence of multiple nucleation events compared to bulk synthetic techniques, due to the reduced volumes available per nanochannel for crystallization.^35^ The decreased number of nucleation events, coupled with slow growth of the MOF crystal, produces large, high aspect ratio crystals that are shaped by the channel dimensions over the course of several days.

### Morphology and characterization of nanoconfined HKUST-1 crystals

Bulk HKUST-1, like many other MOFs, is known to have faceted crystal shapes under ideal growth conditions.^16^ As the crystals grow, surface energy minimization causes the crystal to exhibit different morphologies. Smaller crystals with square faces (associated with the {100} crystal plane) eventually grow into more stable structures with different faceting (*e.g.* the hexagonal or triangular {111} planes).^36^

**Fig. 2:**
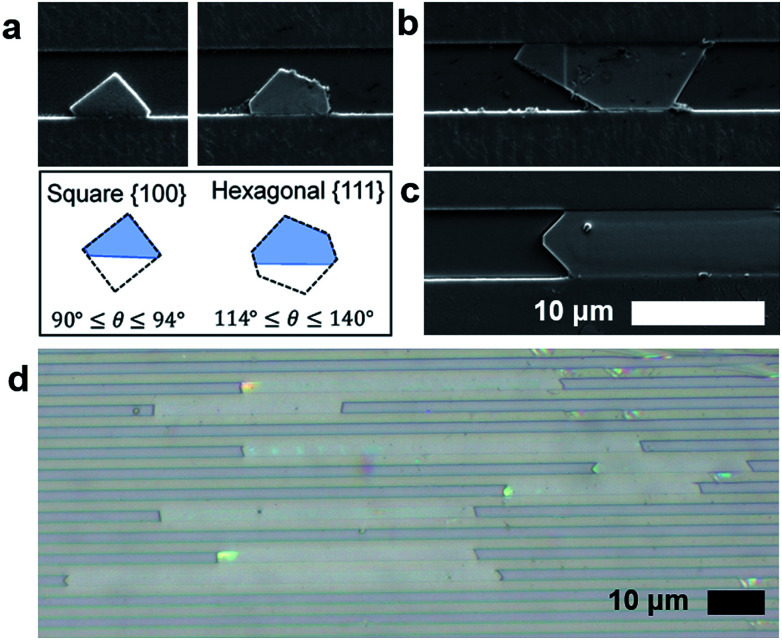
(a) SEM micrographs demonstrating nucleation of square {100} and hexagonal {111} crystal morphology. (b) Nuclei begin to grow larger until inhibited by the channel width and finally (c) crystal growth progresses along the channel length to create high aspect ratio MOF crystals of HKUST-1 in nanochannels. (b) A brightfield optical image of HKUST-1 showing high aspect ratio crystals. Scalebar is 10 μm.

In confined nanofluidic growth, similar faceting is observed and diverse morphologies of crystals in the nanofluidic channels are observed during the growth process (Fig. 2). Observing the population of HKUST-1 crystals that did not grow to fill the channel width, both hexagonal and square prism shaped crystals nucleate in the nanochannels (ESI Fig. 4†). HKUST-1 crystals that grow in the hexagonal shape indicate that the {111} plane is oriented with respect to the nanochannel width, while the square prism shape indicates that the {100} plane is oriented with respect to the nanochannel width. This result agrees with other reported equilibrium morphologies found in thin film and confined HKUST-1, where channel surface chemistry and facet–channel interaction can influence the orientation and morphology of crystals (ESI Fig. 5†).^16,24,37^ Faceting is observed on the crystal edges, and we hypothesize that facets correspond to the slow-growing planes associated with the equilibrium HKUST-1 crystal morphology.^16^

As growth proceeds, we hypothesize that the crystal growth is first confined by the nanochannel height (60–350 nm) and then confined by the nanochannel width (0.5–5 μm), depending on the geometry of each channel (Fig. 2a and b). Following these confinement events, crystal growth occurs along the length of the channel (Fig. 2c). This mode of crystalline growth allows HKUST-1 crystal length to greatly exceed the depth (Fig. 2d).

Assessing the aspect ratio, defined as the ratio of crystal length to depth, we observe that the channels with the shallowest depths (30 nm) produce the largest aspect ratio (ESI Table 3†). The highest aspect ratio observed in this study was approximately 2500 (ESI Fig. 6†), an improvement on previously reported results.^33,38^ Conversely, the larger depth channels (∼300 nm) produce aspect ratios on the order of 200. This finding suggests that depth confinement was significant to producing the highest aspect ratio MOF crystals. The role of channel width on crystallization warrants additional study. However, results suggest that the width dimension does not need to be confined to the same degree as depth (100 nm or less), as widths in the 0.5–1 μm range yield aspect ratios greater than 1000. This is significant, as making and filling channels becomes more difficult as both the depth and width become smaller; hence wider channels that are easier to load with fluid and with nanoconfinement in depth alone are sufficient to form high aspect ratio crystals.

The material properties of the obtained crystals are also consistent with the expected properties of HKUST-1. The final crystallized material is insoluble in ethanol, while the starting materials, trimesic acid and copper nitrate, are soluble. Hence, upon washing the nanochannels after crystallization, the remaining crystal has a thin film morphology and solvent compatibility consistent with that of HKUST-1.

**Fig. 3:**
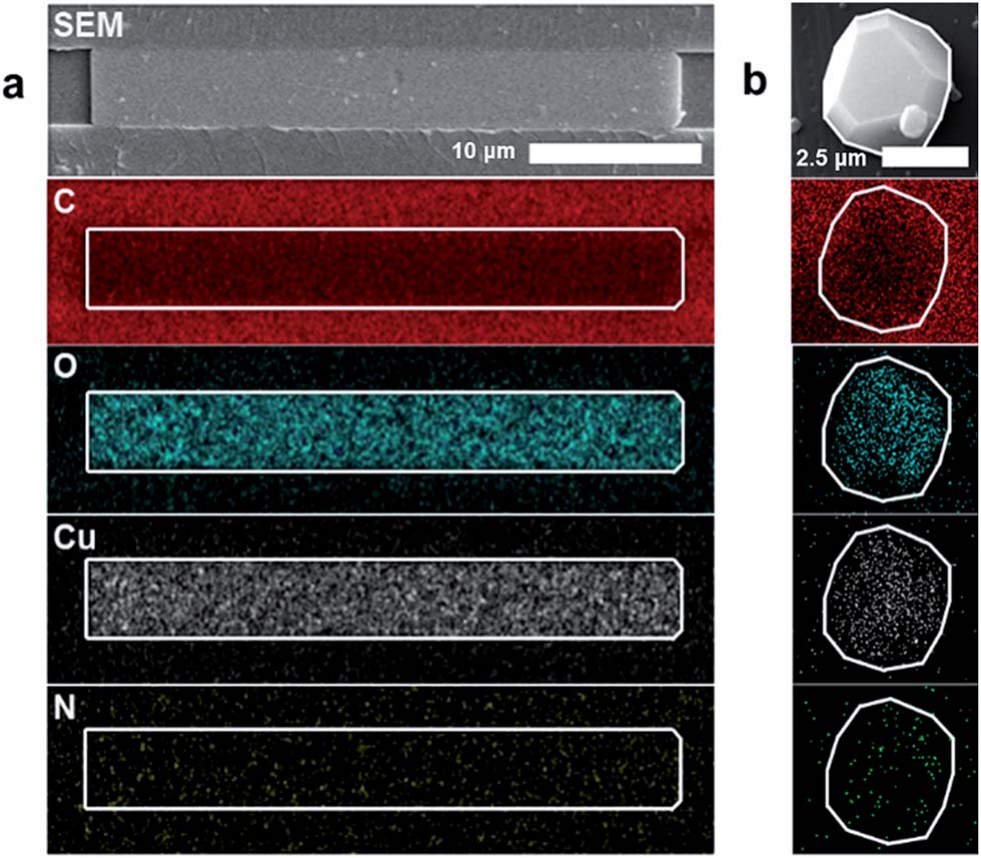
(a) EDS characterization showing the relative intensity of copper (Cu), oxygen (O), carbon (C) and nitrogen (N) compared the COC substrate for a nano-confined HKUST-1 crystal. Scale bar is 10 μm. (b) EDS imaging showing the relative C, O, Cu, N from HKUST-1 drop-casted on COC substrate for a bulk synthesized HKUST-1. Scale bar is 2.5 μm.

Energy dispersive X-ray spectroscopy (EDS) was used to examine the elemental constituents of the crystals in the nanochannels, providing further evidence of HKUST-1. Fig. 3a demonstrates colocalization of copper, carbon and oxygen within the crystal. A decrease in carbon signature of the MOF relative to the COC (a carbon-based polymer) substrate differentiates the HKUST-1 crystals and the nanochannels. The nitrogen signal remains at noise level, while a copper nitrate control shows appreciable nitrogen signal (ESI Fig. 7†). Therefore, EDS results suggest that copper and nitrate ions from the Cu(NO_3_)_2_ hydrate precursor dissociate. The presence of the oxygen signature in the crystal, combined with the absence of the nitrogen signature, indicates that the copper is likely coordinating with the trimesic acid linker. Furthermore, the elemental signatures in the nanoconfined HKUST-1 are consistent with the signals obtained from bulk HKUST-1 control EDS scan (Fig. 3b). Conventional characterization techniques such as BET-based surface area analysis, TEM and X-ray diffraction prove infeasible due to the small amount of material and the sensitivity of the crystals to the degradation using these characterization techniques.

To probe the crystallinity of the HKUST-1 in the nanochannels, cross sections of the nanochannels were prepared for transmission electron microscopy (TEM) using a focused ion beam (FIB), but TEM diffraction does not conclusively yield a pattern representative of HKUST-1 or its precursors. We hypothesize that the FIB process is destructive to the HKUST-1, and can back-deposit organic material into the porous structure (ESI Fig. 8†).^39^ A bulk HKUST-1 crystal subjected to the same FIB/TEM procedure also degrades similarly. More work is required to overcome challenges associated with high energy characterization techniques to develop full understanding of the single-crystal behaviour in nanoconfined environments for this system.

### Loading small molecules into high aspect ratio HKUST-1 crystals

**Fig. 4:**
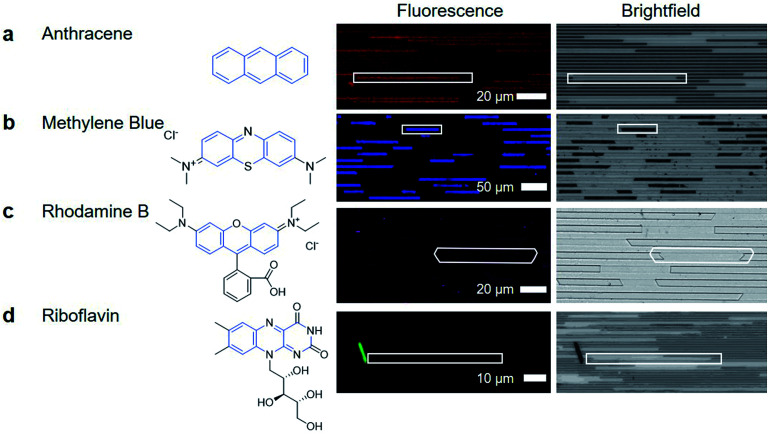
(a) Anthracene is included within the MOF structure, shown by the co-location of fluorescence signals and the transmission brightfield crystal location. (b) Methylene blue, is larger than anthracene and is a charged molecule. Fluorescence and brightfield images show that methylene blue is present in the crystal, and is present in a quantity to make the crystal appear darker in the brightfield image. (c) Rhodamine B, does not appear to enter the MOF. (d) Riboflavin is also excluded from the MOF, with no fluorescence signal detected in regions where HKUST-1 crystals are present in the nanochannels.

After removing the top layer of COC to expose the crystals and nanochannels, pure ethanol was used to wash away unreacted material and debris loosely present on the surface. Despite challenges in characterization, the functional utility of the porous MOF is demonstrated through the capacity of the crystals to uptake and exclude different molecular species. HKUST-1 has two pore apertures of 10 Å and 14 Å. Four different fluorescently active molecules were selected as candidate guest molecules. These molecules were selected because they have similar conjugated cores, with anthracene being the simplest, containing three aromatic rings. The other molecules have larger molecular size and greater chemical complexity due to the introduction of additional functional groups or charges (Fig. 4).

To determine if the guest molecules can enter HKUST-1 selectively, anthracene, methylene blue, riboflavin or rhodamine B were dissolved in ethanol at their saturation concentration at room temperature. The nanochannels were then immersed in the prepared solution and left overnight to allow sufficient time for diffusion to occur before imaging, although diffusion in MOFs has been shown to occur within minutes.^40^Fig. 4 summarizes the results of molecular inclusion of methylene blue and anthracene, with the exclusion of rhodamine B and riboflavin.

Previous studies have shown that the MOF ZIF-8 can be loaded with anthracene while still preserving the characteristic fluorescent emission of anthracene.^41^ We observed that anthracene can also penetrate the framework of HKUST-1 (Fig. 4a). The presence of anthracene seems to have little effect on the appearance of the HKUST-1 crystal; the brightfield image of the anthracene loaded HKUST-1 does not show any significant change. The methylene blue loaded HKUST-1 (Fig. 4b) appears much darker in the brightfield image, showing that there is decreased light transmission through the methylene blue loaded HKUST-1 crystals.

The dark blue color of methylene blue allows for bright-field optical characterization (ESI Fig. 9†) while the fluorescence allows for sensitive measurements to be made while minimizing signal from the COC and HKUST-1. Both techniques show that methylene blue is located inside the crystal, indicating that the formation of a high aspect ratio and porous HKUST-1 crystal was achieved in the nanochannels (Fig. 4b). The capacity for a MOF to uptake these materials is not unique; previous reports have demonstrated methylene blue diffusion into the pores of HKUST-1.^42,43^ However, the advantage of nanoconfined MOF crystallization is the potential for localized and selective enrichment of analytes to enhance detection selectivity.

The nanoconfined diffusion path length from the top to the bottom of the channel allows for rapid equilibration of the analytes with minimal concentration gradient across the channel depth. The uniformity of the thin film and the large aspect ratios also allows for clear optical or fluorescent detection of analytes due to the uniform optical path length (Fig. 4, ESI Fig. 10†).

Fig. 4c shows that rhodamine did not enter the MOF at a detectable concentration. The signal in the image is from small crystallites of rhodamine B deposited on the channel surface, or is noise during the imaging. Finally, riboflavin is not detected within the HKUST-1 crystals either. The images in Fig. 4d show a large crystal, which is hypothesized to be riboflavin that was deposited as ethanol evaporated during the drying process, causing riboflavin to precipitate out of solution. Therefore, the porous MOF has the ability to be used for small organic molecule separations (*e.g.* allowing methylene blue to pass through the crystal while keeping rhodamine B out)^44^ Through this, the utility of high aspect ratio nanochannel crystallization of a MOF is made clear. It can serve as a platform for capture and enrichment of a material, followed by subsequent detection. This is feasible even when there is only a small amount of material available, such as for biomarker detection, which requires sensitivity and specificity for detection, despite limitations of small working volumes.^45,46^

Additional studies for crystallization were performed in the nanoconfined channels using the precursor for methyl ammonium lead iodide (MAPbI_3_), a perovskite structure frequently used for solar cell applications. While confined crystallization did occur within the nanochannels (ESI Fig. 11†), the optimal concentration and crystallization conditions required to achieve high aspect ratio and single crystalline growth are still being explored.

## Conclusion

We present the first report on crystallization of HKUST-1 in parallel arrays of nanoconfined channels for studying crystal morphology and characterizing the crystals for selective uptake of small organic molecules. Crystal morphology, porosity, elemental mapping and fluorescence microscopy techniques indicate that oriented, high aspect ratio, crystalline domains of HKUST-1 were formed in nanochannels. Moreover, aspect ratios greater than those previously reported for MOFs were obtained, reaching 2500. This work provides the foundation for a range of fundamental studies involving MOFs and other two component reaction-crystallization systems with applications in understanding thin film growth, nucleation and growth, diffusion kinetics, and in sensing guest molecules in MOFs.

With a well-defined and optically accessible growth region, we are able to observe the nucleation and growth of individual HKUST-1 crystals using readily available optical techniques. Future studies will focus on controlling and understanding crystallization kinetics for tunable crystal aspect ratios. By adjusting concentration and temperature, it is possible to bias a system towards nucleation or growth dominated regimes.^47,48^ The nanofluidic device studied here can be further developed with patterning and dynamic flow control for increased control over crystal growth direction and morphology.

Large crystal domains of HKUST-1 allow for lower impact of grain boundaries for highly efficient sensing and separation. In future work, fluid flow can also be introduced into the nanochannels after the creation of high aspect ratio MOF crystals to achieve spatial and temporal control over the introduction of guest molecules (analytes).

There is a rich field of future work possible with this technology, as the platform is ideal for integration with many applications (including sensing and separations). We intend to study the diffusion of small fluorescent molecules out of the HKUST-1 crystal in the nanochannels. This is important for probing the mechanism of diffusion in MOF materials, related to zeolites and other mesoporous materials where surface interactions are significant for material performance. This work will be extended in the future to molecular biomarkers that are usually present at dilute levels within biofluids prior to significant disease progression, thereby requiring significant levels of enrichment before detection may be possible.^49^ In this context, MOFs designed for selectivity to particular molecules and synthesized within a nanoconfined platform to form high aspect ratio, defect-free crystals, can be coupled to a microfluidics network to enable selective analyte enrichment and detection. Finally, separations and sensing applications can leverage the high aspect ratio and nanometer thickness of the crystals grown in this study.

## Experimental

### Materials

#### MOF precursor, fluorophores, and other chemicals

MOF precursor was composed of dimethyl sulfoxide (DMSO, 99.9%), copper(ii) nitrate hemi(pentahydrate) (Cu_2_(NO_3_)·2.5H_2_O, 98%) and trimesic acid (H_3_btc, 95%), sourced from Sigma Aldrich. Fluorophores were dissolved in pure ethanol (Koptek, 100%). Fluorescent molecules used in this work include methylene blue (certified by the Biological Stain Commission, Sigma Aldrich), rhodamine B (suitable for fluorescence, Sigma Aldrich), anthracene (analytical standard, Sigma Aldrich), and riboflavin (>98%, Sigma Aldrich) were used for post synthetic visualization of porosity. All chemicals were used as received.

#### Nanochannel fabrication and materials

Silicon wafers (University Wafer) were patterned by electron beam lithography and reactive ion etching to fabricate a master mold containing a network of nanochannels connected to microchannels.^45,50^ This master mold was used to fabricate nanochannels by hot emboss imprinting on 175 μm thickness cyclic olefin copolymer or COC substrates (Microfluidic-Chip Shop: TOPAS), by using a working stamp polymer M7+IGRACURE 2022 (EV group, Inc.) as an intermediary stamp to prevent damage to the master mold.^51^ The nanochannels were then bonded to a 175 μm thick COC cover slip (Microfluidic-Chip Shop: TOPAS), which was drilled for inlet and outlet reservoirs that lead to the microfluidic channels.

### Methods

#### HKUST-1 crystallization

Precursor solution was prepared with Cu(NO_3_)_2_·2.5H_2_O, trimesic acid and DMSO following the general procedure outlined by Ameloot *et al.*^24^ A typical precursor was made by completely dissolving 1.22 g Cu(NO_3_)_2_·2.5H_2_O in 5 mL of DMSO, then dissolving 0.58 g of trimesic acid in the same solution. The final solution volume was 5.625 mL, yielding metal and linker concentrations of 0.93 M and 0.49 M, respectively. Channels were loaded by placing a small quantity (5 μL) of the precursor solution on the channel ports. Capillary action was observed to wick precursor into the nanochannels immediately after placement. Excess fluid at channel ports was gently removed using a Kimwipe. Channels were imaged after loading to determine initial loading efficiency. HKUST-1 crystallization was induced by evaporating solvent from the channels. A vacuum oven could be used to control the pressure to increase evaporation rate and enhance crystallization.

#### Fluorescent molecule loading

To study molecular uptake in the HKUST-1 crystals, nanochannels containing crystals were immersed in a solution of pure ethanol and a fluorophore (either anthracene, methylene blue, rhodamine B or riboflavin). These solutions were prepared at saturation concentration. The channels with crystals soaked in this solution for 24 hours. To allow sufficient time for the molecule to penetrate the MOF framework and fill the pores. The channels were briefly rinsed with ethanol, dried in ambient conditions, and imaged using a confocal microscope.

#### Crystal characterization

A Zeiss Scope A.1 optical microscope with an Axiocam 503 Color camera was used to image crystals within the nanochannels. A Zeiss 780 NLO confocal laser scanning microscope was used for fluorescence imaging of crystals after soaking with the fluorescent molecules. The fluorescence was measured using an external transmission NDD PMT detector.

A Quanta 650 scanning electron microscope (SEM) (5 kV, spot size 4, Everhart–Thornley detector) was used to collect morphological data from crystals in the nanochannels. Energy dispersive X-ray spectroscopy (EDS) (18 kV beam, Oxford Instruments, X-Max^N^ 80) was used analyze elemental composition of crystals in delaminated channels. Channels were prepared for these processes by sputter coating them with a protective layer (∼20 nm thick) of gold-palladium using a Precision Etching and Coating System (Model 682 PECS).

## Conflicts of interest

The authors declare no conflicts.

## Supplementary Material

NA-001-C9NA00254E-s001
