# Supplementary material for: Crystallization of high aspect ratio HKUST-1 thin films in nanoconfined channels for selective small molecule uptake
Source: Nanoscale Adv. 2019 Jun 12;1(8):2946–52. doi: 10.1039/c9na00254e (PMC9418888; doi:10.1039/c9na00254e)
Supplement: NA-001-C9NA00254E-s001 [file NA-001-C9NA00254E-s001.pdf]

Electronic Supplementary Material (ESI) for Nanoscale Advances.
This journal is © The Royal Society of Chemistry 2019

Supporting Information

Crystallization of High Aspect Ratio HKUST-1 Thin Films in Nanoconfined Channels for Selective Small Molecule Uptake

Stephanie Guthrie[a†], Luke Huelsenbeck[a†], Armita Salahi[b], Walter Varhue[b], Natalie Smith[a], Xiaohan Yu[c], Lucy U. Yoon[a], Joshua J. Choi[a], Nathan Swami[b], Gaurav Giri[a*]

a. *Department of Chemical Engineering, University of Virginia, Charlottesville, Virginia 22904, United States*
b. *Department of Electrical and Computer Engineering, University of Virginia, Charlottesville Virginia 22904, United States*
c. *Department of Chemical Engineering, Tsinghua University, Beijing, 100084, China*

† These authors contributed equally to this work
*Corresponding Author

Nanochannel Device Fabrication:

The nano-channel devices are fabricated using a Hot-Embossing Nano Imprint Lithography method (HE NIL). This method was chosen as it can reliably replicate the nano-depth features of an expensive and hard to fabricate silicon master in an inexpensive wafer made of the thermoplastic cyclic olefin copolymer (COC) (TOPAS; microfluidic-chip shop). The silicon master is fabricated by etching the nano-channel features into a polished <100> silicon wafer using a standard photolithography and reactive ion etching (RIE) process. Features from the silicon master are then negatively transferred to a soft working stamp, which is comprised of a thin layer of UV curable elastomeric polymer (Working stamp polymer M7 + IRGACURE 2022; EV group) affixed to a glass wafer (Borfloat 33). This is done through a micro-molding process by compressing uncured elastomer between the silicon master and a blank glass wafer treated with an adhesion promoter (Working stamp primer KR513; EV Group) allowing the uncured polymer to conform to the nano-features of the master. Metal spacers are used to control the gap between the master and glass wafer during compression, ensuring that a uniform elastomer layer forms. The working stamp material is cured through UV exposure, transferring a negative of the nano-channel features to the thin polymer layer now affixed to the glass wafer. The soft stamp is then demolded from the silicon master, which had been treated with an anti-stiction layer (Trichloro(1H,1H,2H,2H-perfluorooctyl)silane) prior to molding. This soft stamp is then used to imprint the nano-channel features into a COC wafer using a HE NIL method. Using a hot embosser NIL tool (EVG 510, EV group), a blank COC wafer is embossed with the soft stamp for 20 min at 160 C and 6000 N of force. When heated to its glass transition temperature, the COC enters a viscus state, allowing it to conform under pressure to the semi-rigid features of the soft stamp. After the COC cools and sets, it can be demolded from the soft stamp resulting in a high-fidelity replication of the nano-channel features from the silicon master. Finally, the imprinted nano-channels are encapsulated by a thin COC coverslip through fusion bonding. An inlet and outlet are drilled through a 175 µm thick COC coverslip and then aligned to either side of the imprinted nano-channels. The coverslip is then bonded to the COC wafer using the hot embosser tool by applying 7450N of force at 130C for 30 minutes.

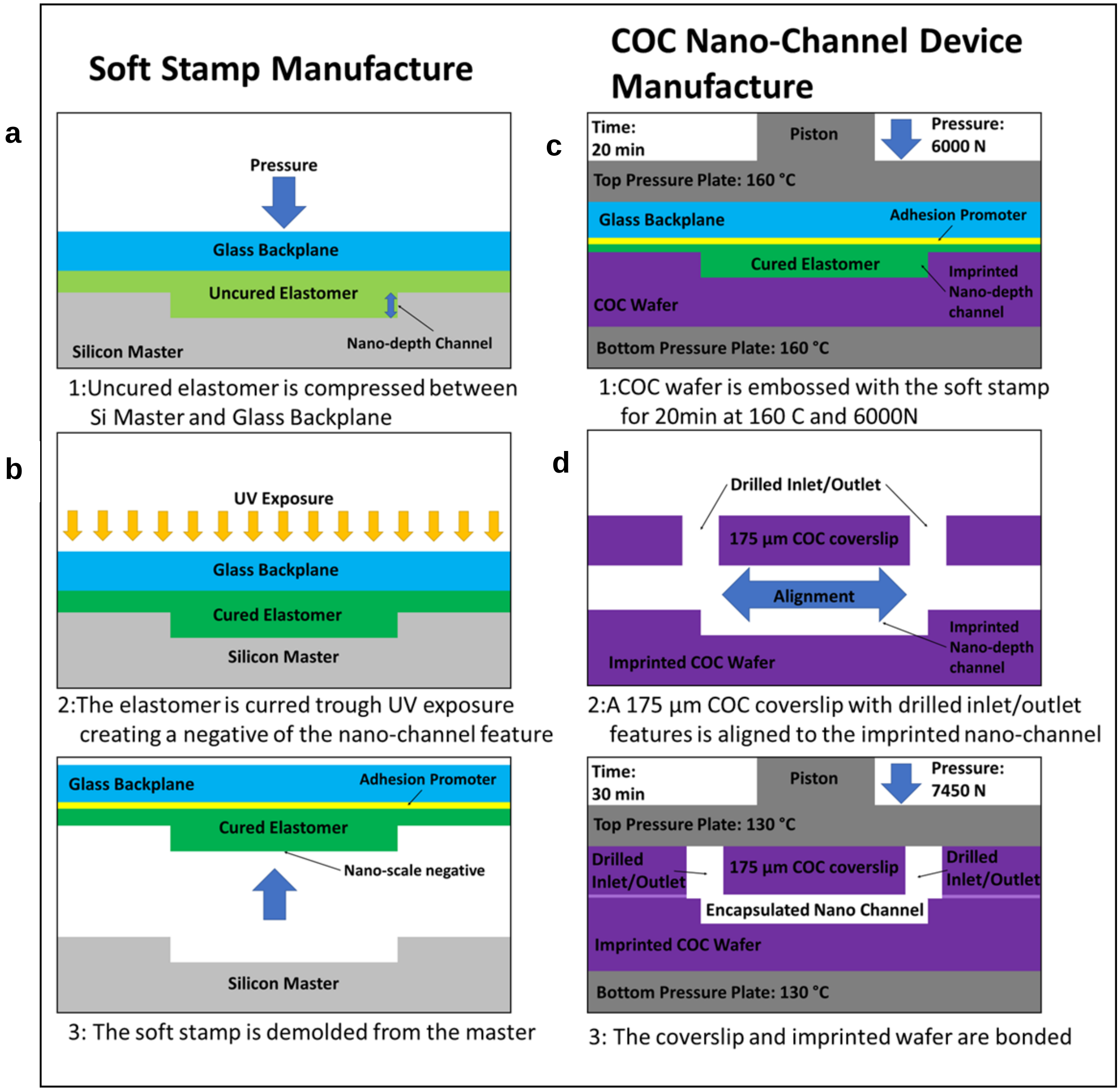

SI Figure 1: The cyclic olefin copolymer (COC) nanofluidic device is fabricated from a series of lithography techniques. a) First, a silicon master is etched with the desired channel profiles (length width and depth). b) The silicon patterning is transferred to an elastomer that has the ability to transfer patterning to the COC device. c) using heating and pressure, the elastomer pattern is pressed into COC (175 or 1000 µm thicknesses used) d) nanoscale features are enclosed by another COC coverslip with inlet and outlet holes prepared for solution loading.

Additional Nanochannel Characterization

The fabricated channels are located on a COC wafer, 4” in diameter. This contains multiple arrays, for higher throughput experimentation (**Figure S3**). Figure S3c shows, further, that the arrays were characterized for controlled length, width and depth.

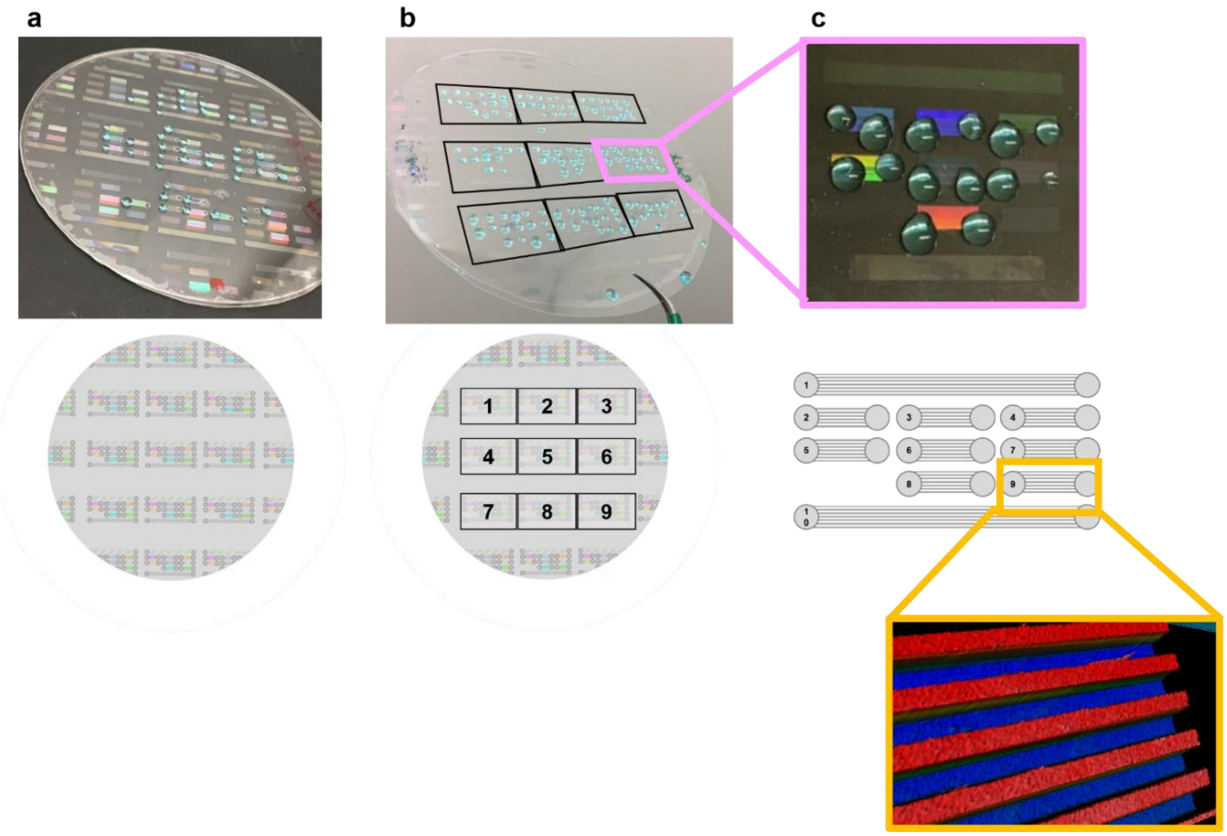

SI Figure 2: a) Overview of the fabricated nanofluidic device b) The device contains nine complete sets of nanochannels, with each set containing c) 10 channels, each with a specified length, depth and width.

The target and actual achieved specifications for channel fabrication were measured and recorded, shown in SI table 1.

Table S1: Nanochannel specifications for each of the 10 channels in the array.

Channel	Width (µm)	Target width during fabrication (µm)	Depth (nm)
1	5	5	285
2	0.72	0.75	100
3	0.5	0.500	<60
4	2	2	325
5	0.76	0.75	60
6	0.43	0.460	<30
7	5	5	325
8	1	1	100
9	10	10	325
10	18.25	20	Not Measured

The depth was measured using a Zygo white light interferometer, while channel width was visible under SEM. Table S2 outlines the two examples of the measurements and recorded channel depth.

Table S2: Width and depth measurements taken from SEM and a white light interferometer. This demonstrates the capability creating crystals with highly controlled width and depth through nanofluidic confinement. SEM images show the sharp edge features for high quality control over morphology.

Channel Type	Depth Measurements (Zygo interferometer measurements)	Width (from SEM) characterization
Thin	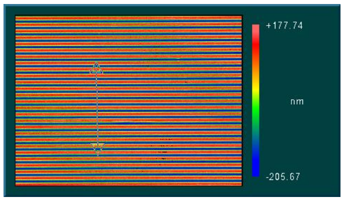 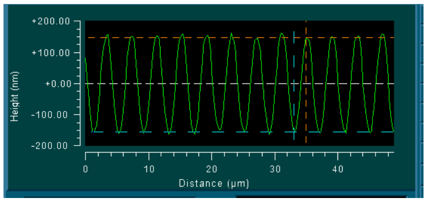 325 nm	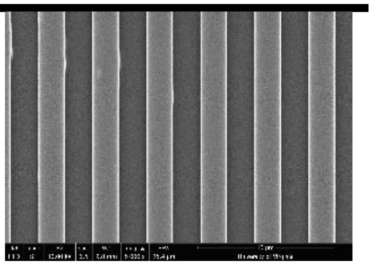 2 µm
Wide	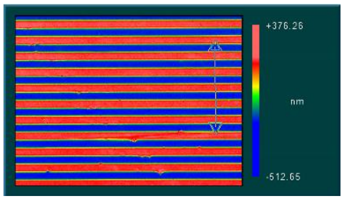 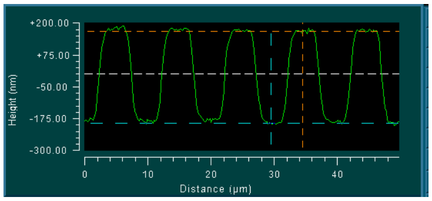 325 nm	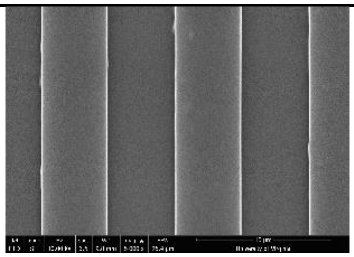 5 µm

Channel Loading

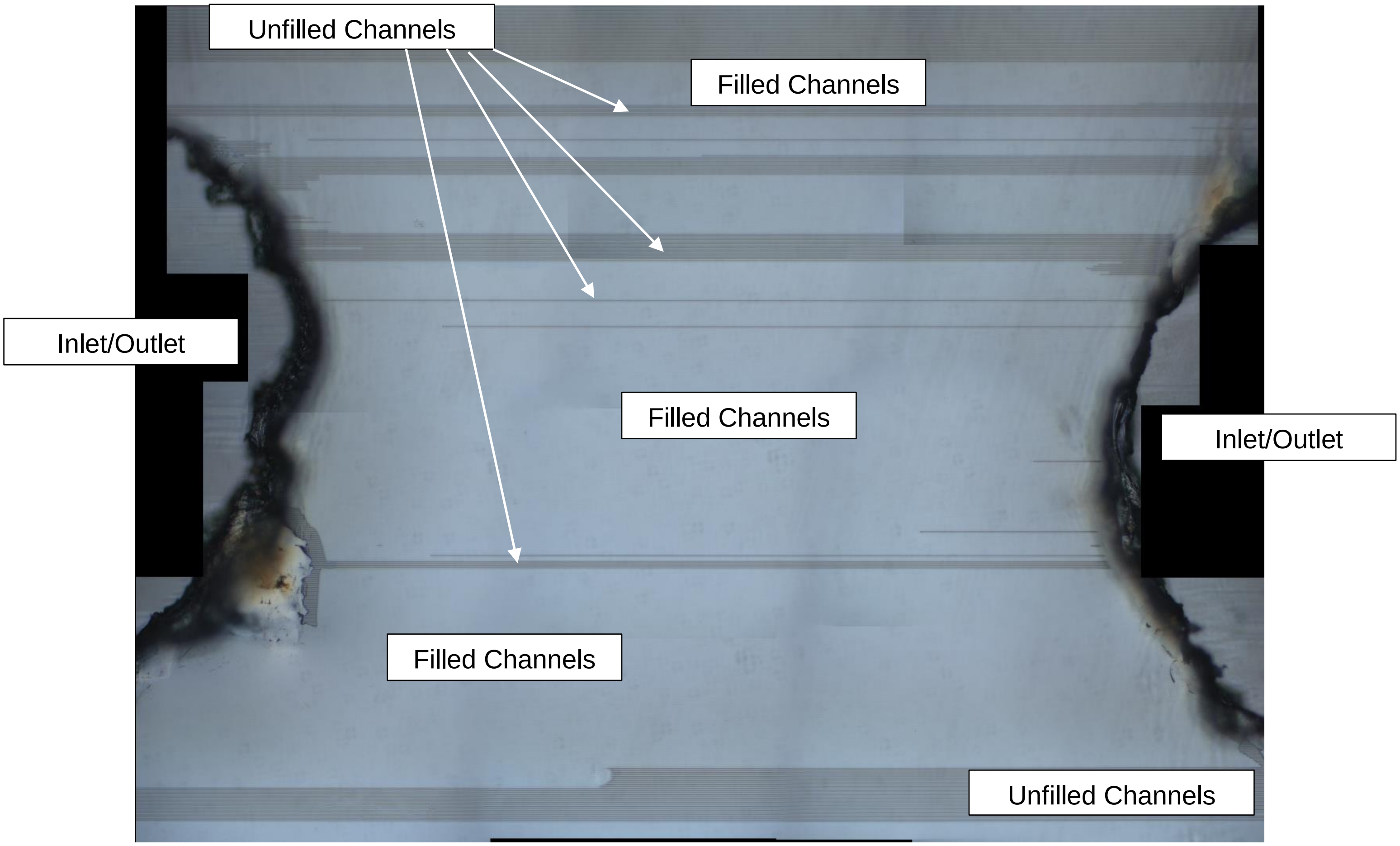

SI Figure 3: Optical brightfield stitched image of nanochannel with channel ports shown on either end. Light color are channels loaded with HKUST-1 precursor solution, dark channels are unloaded. 20X microscope images are stitched together using imageJ stitching tools. This allows for observation of the whole nanochannel region where crystallization can occur.

Calculating Maximum Theoretical Yield of Crystal for a Nanochannel
Reaction Scheme for HKUST-1 Formation (ACS sustainable chem, 2017, 5, 7887-7893)

3 $Cu(NO_3)_2$ + **2** BTC → **1** HKUST + **6** HNO_3

HKUST-1 molecular weight: $604.87\,\frac{g}{mol}$

HKUST-1 Density $0.35\,\frac{g}{cm^3}$

Precursor solution (0.49 M BTC, 0.93 M $Cu(NO_3)_2$

BTC (linker) is the limiting reactant.

The maximum theoretical volume yield of HKUST-1 is calculated as follows:

$$0.49\left[\frac{mol\ linker\ consumed}{L\ precursor}\right]*\frac{1}{2}\left[\frac{mol\ HKUST1\ formed}{mol\ linker\ consumed}\right]=0.245\left[\frac{mol\ HKUST1\ formed}{L\ precursor}\right]$$

$$0.245\left[\frac{mol\ HKUST1\ formed}{L\ precursor}\right]*604.87\left[\frac{g\ HKUST1}{mol\ HKUST1}\right]=148.19\left[\frac{g\ HKUST1}{L\ precursor}\right]$$

$$148.19\left[\frac{g\ HKUST1}{L\ precursor}\right]*\frac{1}{10^{12}}\left[\frac{L}{picoliter}\right]=1.48*10^{-10}\left[\frac{g\ HKUST1}{picoliter\ precursor}\right]$$

$$1.48*10^{-10}\left[\frac{g\ HKUST1}{picoliter\ precursor}\right]*\frac{1}{0.35}\left[\frac{cm^3 HKUST1}{g\ HKUST1}\right]$$
$$=4.22*10^{-10}\left[\frac{cm^3\ HKUST1}{picoliter\ precursor}\right]$$

$$0.422\ \frac{picoliters\ HKUST1}{picoliter\ precursor}$$

If the precursor solution completely fills the channel, then 100% channel loading efficiency is achieved. After crystallization occurs, if the reaction yield is 100%, then only 42% of the channels will be filled with crystal volume (0.42 pL HKUST-1 per pL of precursor). Therefore, complete channel loading will not achieve complete crystallization fill in the nano confined channels.

HKUST-1 Crystal Morphology

Representative SEM images of small, low aspect ratio crystals with hexagonal and square faceting.

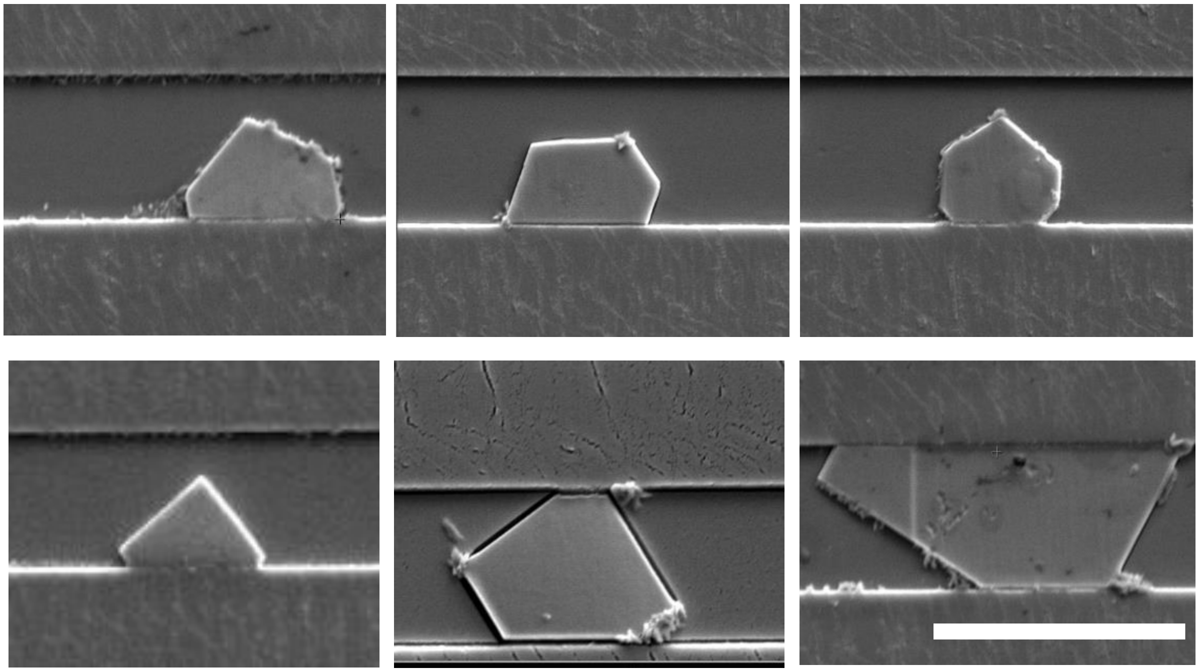

SI Figure 4: Hexagonal and square crystal morphology demonstrating crystal orientation relative to the channel bottom. Crystals do not appear to exhibit orientation effects with respet to the channel walls. Scale bar is 10 µm.

Bulk HKUST-1 Crystal Morphology

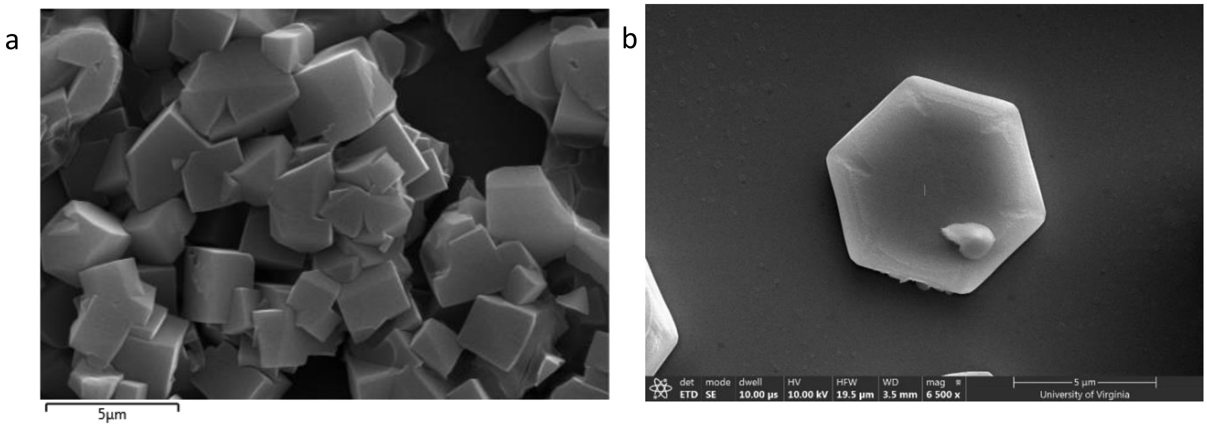

SI Figure 5: Bulk HKUST-1 crystal morphologies. Here we observe (a) cubic and (b) hexagonal morphology.

High Aspect Ratio Crystals in Nanochannels:

The aspect ratio determined here and throughout the publication is defined as ratio of the largest dimension to the smallest dimension. With the crystal filling being space filling, the crystal depth is the same as the channel depth.

$$Aspect\ Ratio = \frac{Crystal\ Length}{Crystal\ Depth}$$

Images were captured using a brightfield view on the Zeiss Axio A1 Scope, as discussed in materials and methods.

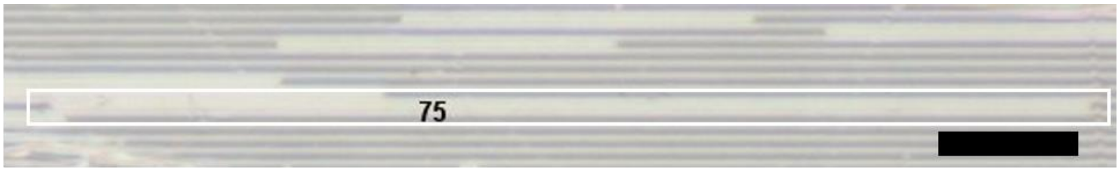

SI Figure 6: Example of a high aspect ration crystal, formed in channel 6. The crystal length was measured to be 75 μm, and the depth 0.03 μm, L/D = 2500. Scale bar = 10 μm

Table S3: Channels have controlled depth (~100-350 nm) and width (~1-5 um) differences to demonstrate the ability to influence aspect ratio, where aspect ratio is defined as longest dimension compared to the shortest dimension. Table is sorted by increasing channel depth, demonstrating that the channel confinement is significant in creating the high aspect ratio crystals.

Channel Depth (nm)	Channel Depth (μm)	Channel Width (μm)	Max Crystal Length (μm)	Aspect Ratio
30	0.03	0.425	75	2500
60	0.06	0.5	111	1850
60	0.06	0.765	132	2200
100	0.1	0.715	144	1440
100	0.1	1	108	1080
285	0.285	5	52	182
325	0.325	2	143	440
325	0.325	5	103	316

Characterization of Copper Nitrate in Nanochannels

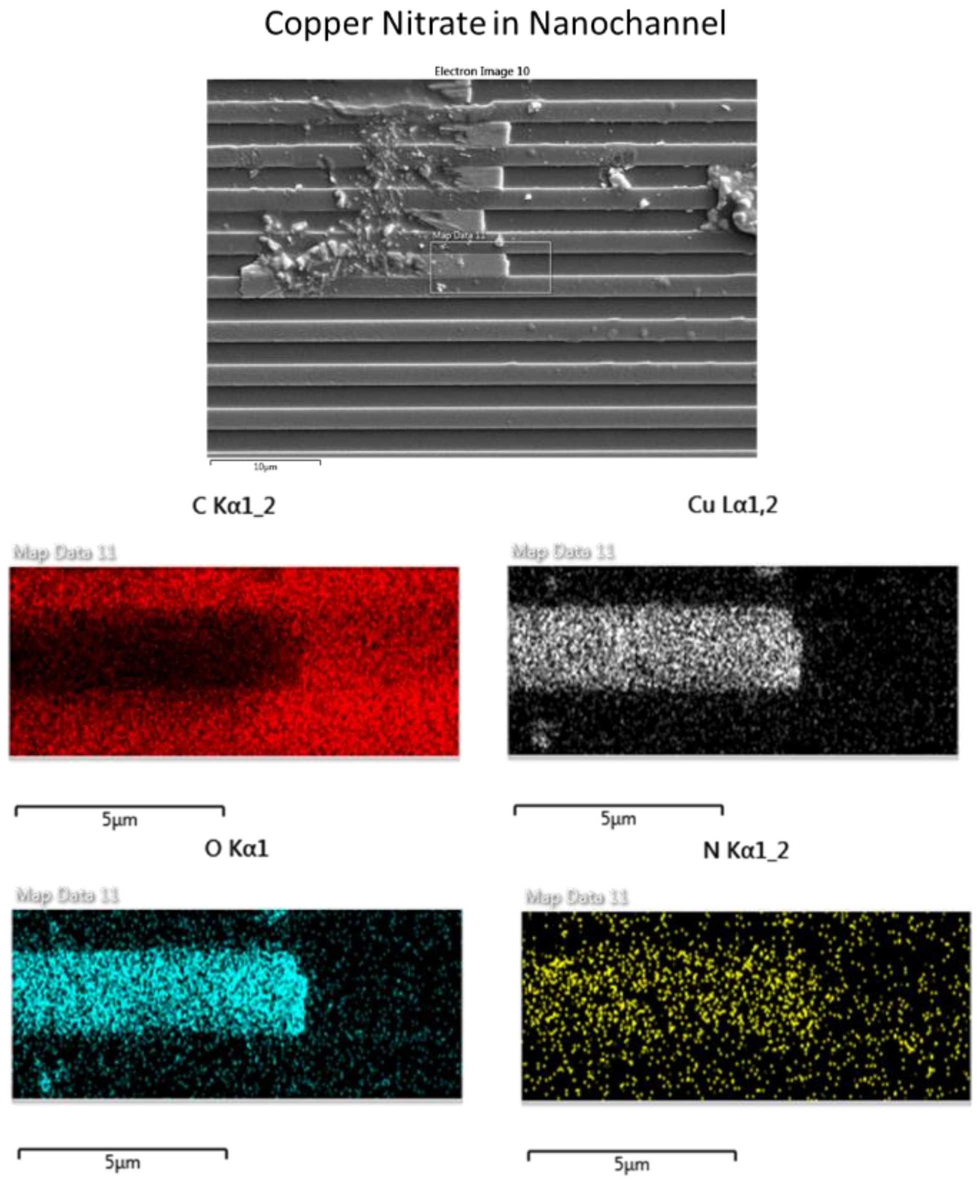

SI Figure 7: Copper nitrate crystallized in the nanochanels, showing low carbon signal, high copper, oxygen and nitrogen signals. This demonstrates that in the MOF, nitrogen signal is diminished due to dissociation of the nitrate form the copper (II). HKUST-1 crystallized in nanochannels s most similar to the bulk EDS signal, not similar to copper nitrate, suggesting successful creation of HKUST-1 in the nanochannels.

Focused Ion Beam (FIB) damage to HKUST-1 in a Nanochannel

Channel and crystal cross sections of 100 nm were prepared from delaminated channels using a Helios Dual Beam FIB G4 UC. FIB milling was performed with a Ga ion beam current of 2.5-9.3 nA at 30 kV. This process deposited additional layers of protective material, consisting of gold-palladium or carbon and platinum. High resolution transmission electron micrographs (HRTEM) and diffraction patterns were taken of 100 nm thick cross section samples using a FEI Titan.

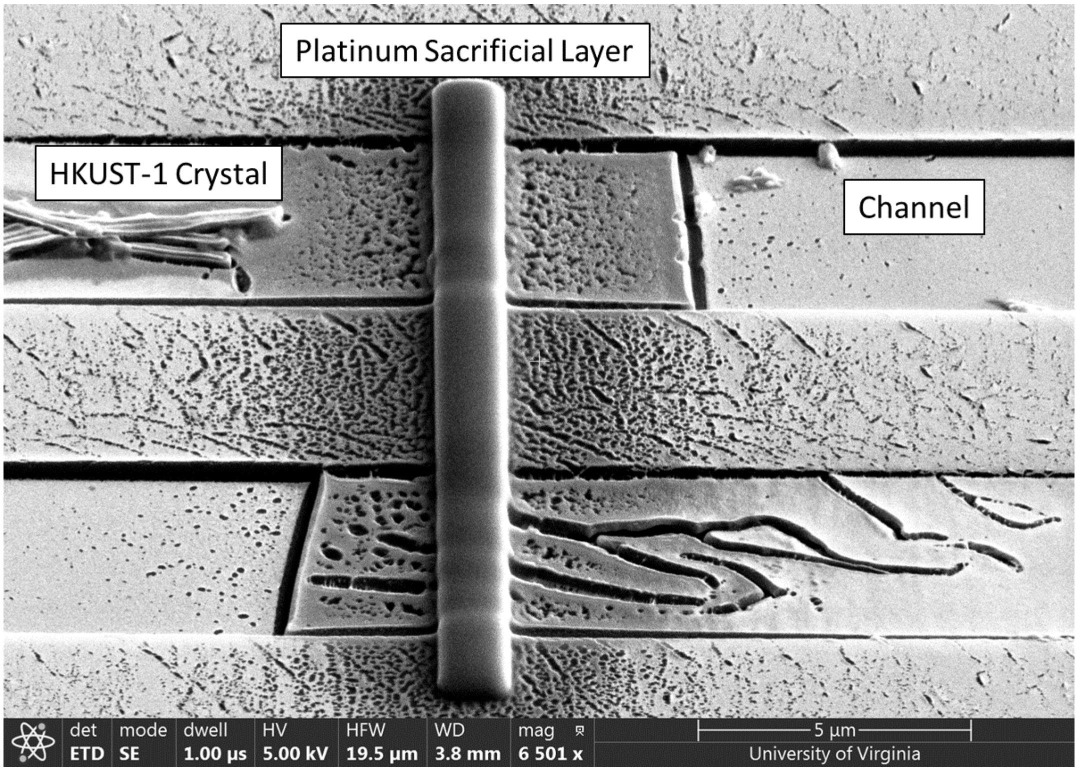

SI Figure 8: During section and harvesting of crystals from nanochannels, the material is damaged. The focused ion beam is high in energy and can degrade the metal organic framework, inhibiting TEM collection of diffraction.

Small Molecule Uptake in HKUST-1 Crystals

Methylene blue, a fluorescing dye, was loaded in the high aspect ratio crystals. The successful loading with this material are obvious, and can be seen visibly or under brightfield microscopy.

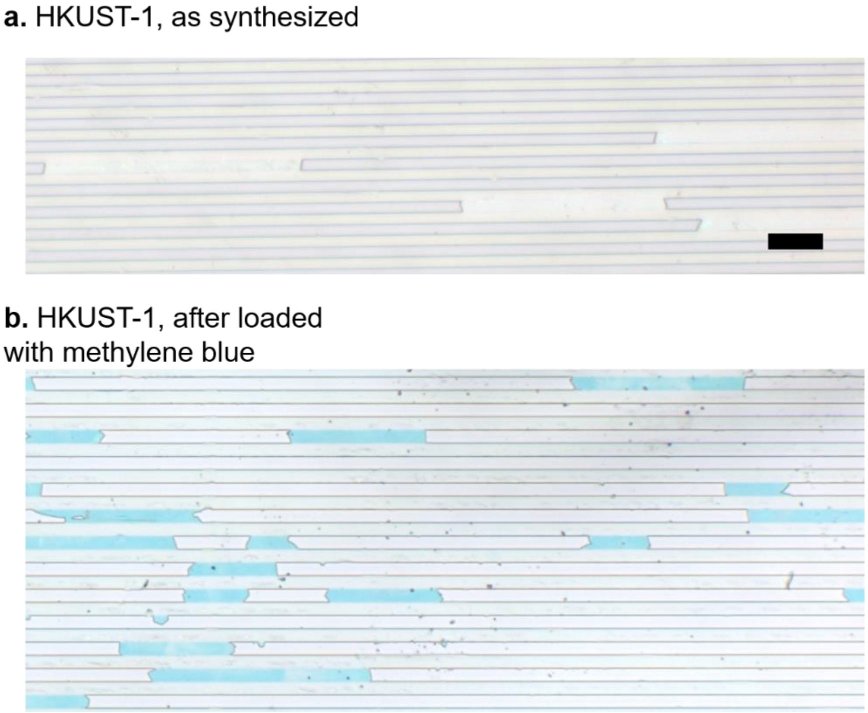

SI Figure 9: a) HKUST-1 crystals in nano-confined channels and b) crystals after being loaded with methylene blue. The crystals are visibly a different color using brightfield imaging.

Fluorescence

Images of bulk HKUST-1 crystals (those that were formed outside of the confinement region of nanochannels, such as the fluid inlet and outlet) are shown in SI figure 7. These crystals demonstrate the same loading behavior as the confined high aspect ratio crystals.

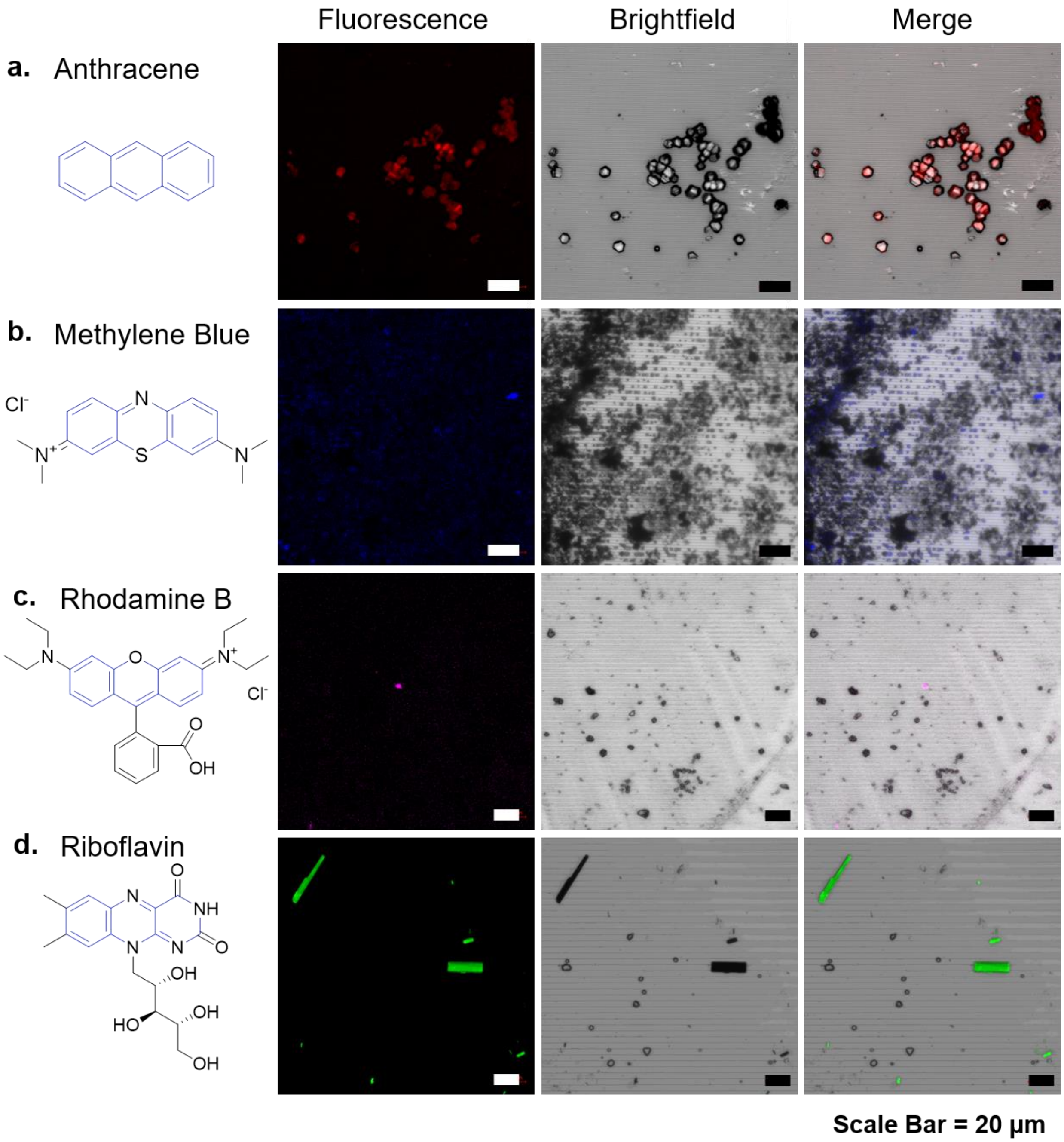

SI Figure 10: Crystals formed on the channels, that can be considered as bulk HKUST-1. a) anthracene, b) methylene blue c) rhodamine B and d) riboflavin. The fluorescence is not located within the HKUST-1 crystals for riboflavin and rhodamine B. Anthracene and methylene blue demonstrate capacity for molecular uptake, with fluorescence appearing in the bulk crystals.

$MAPbI_3$ Crystallization

The perovskite unit cell is isotropic, indicating that the index of refraction is similar in all directions. Therefore, this perovskite structure should not be optically active under cross polarized light. Here optical activity of some regions was observed, indicating the material was anisotropic and likely not perovskite. However, some regions showed no optical activity and color similar to that of the expected perovskite. Similar challenges for characterization of this material were present, including stability issues in atmosphere. This made removing the channel cover difficult without rapidly degrading the crystal.

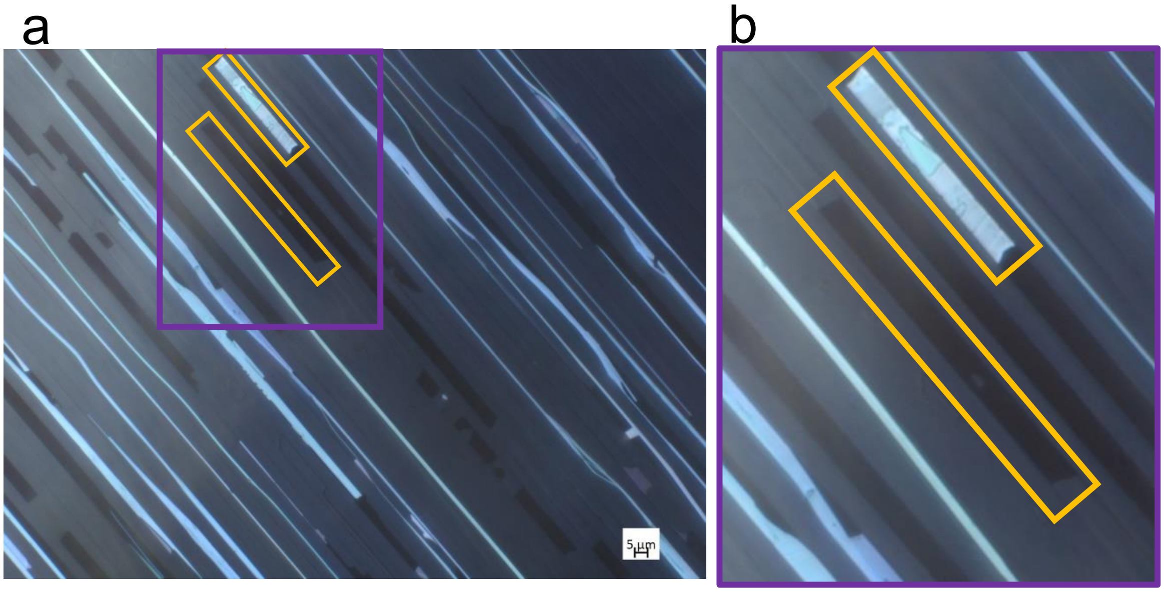

Figure S11: (a) $MAPbI_3$ crystals in the nanochannel. Methylammonium lead iodide ($MAPbI_3$) crystallization was attempted in the nanochannel device. (b) Magnified picture of crystals showing an optically active (bright) crystal near a dark crystal that is not optically active, presumably the intended perovskite material.
